# HDAC7 is a potential therapeutic target in acute erythroid leukemia

**DOI:** 10.1038/s41375-024-02394-5

**Published:** 2024-09-15

**Authors:** Wenyu Zhang, Keita Yamamoto, Yu-Hsuan Chang, Tomohiro Yabushita, Yangying Hao, Ruka Shimura, Jakushin Nakahara, Shiori Shikata, Kohei Iida, Qianyi Chen, Xichen Zhang, Toshio Kitamura, Susumu Goyama

**Affiliations:** 1 Division of Molecular Oncology, Department of Computational Biology and Medical Sciences, Graduate School of Frontier Sciences, The University of Tokyo, Tokyo, Japan; 2 Division of Molecular Pharmacology of Malignant Diseases, Graduate School of Pharmaceutical Sciences, The University of Tokyo, Tokyo, Japan; 3 Institute of Biomedical Research and Innovation, Foundation for Biomedical Research and Innovation at Kobe, Kobe, Japan

**Keywords:** Targeted therapies, Cancer models, Acute myeloid leukaemia

## Abstract

Acute erythroleukemia (AEL) is a rare subtype of acute myeloid leukemia with a poor prognosis. In this study, we established a novel murine AEL model with *Trp53* depletion and ERG overexpression. ERG overexpression in *Trp53*-deficient mouse bone marrow cells, but not in wild-type bone marrow cells, leads to AEL development within two months after transplantation with 100% penetrance. The established mouse AEL cells expressing Cas9 can be cultured in vitro, induce AEL in vivo even in unirradiated recipient mice, and enable efficient gene ablation using the CRISPR/Cas9 system. We also confirmed the cooperation between ERG overexpression and TP53 inactivation in promoting the growth of immature erythroid cells in human cord blood cells. Mechanistically, ERG antagonizes KLF1 and inhibits erythroid maturation, whereas TP53 deficiency promotes proliferation of erythroid progenitors. Furthermore, we identified HDAC7 as a specific susceptibility in AEL by the DepMap-based two-group comparison analysis. HDAC7 promotes the growth of human and mouse AEL cells both in vitro and in vivo through its non-enzymatic functions. Our study provides experimental evidence that TP53 deficiency and ERG overexpression are necessary and sufficient for the development of AEL and highlights HDAC7 as a promising therapeutic target for this disease.

## Highlights

- *TP53* deficiency and ERG overexpression collaboratively induce the development of AEL.
- HDAC7 is a promising and selective therapeutic target in AEL.

## Introduction

Acute erythroleukemia (AEL) is a rare and aggressive subtype of acute myeloid leukemia (AML) involving uncontrolled proliferation of erythroid precursors leading to the accumulation of immature and abnormal red blood cells in the bone marrow and peripheral blood [1]. AEL has traditionally been recognized as having two subtypes: the more common erythroid/myeloid leukemia (EML), defined by the presence of increased erythroid cells and myeloid blasts; and the very rare pure erythroid leukemia (PEL), characterized by the expansion of immature erythroid cells only. Although EML is no longer considered a distinct entity, PEL is still recognized as a distinct variant of AML in both the 2016 and 2022 World Health Organization (WHO) classification systems. It is now widely recognized that true AEL, characterized primarily by immature erythroid proliferation, is often associated with highly complex cytogenetic alterations and biallelic loss of TP53 function [2].

AEL is typically a disease of older adults and has a very poor prognosis with a median survival time of typically less than 6 months. *TP53* mutations/deletions are often accompanied by gains and amplifications involving the *JAK2/EPOR/MPL* genes and *ERG/ETS2* in AEL [3, 4]. *JAK2*, *EPOR*, and *MPL* are the genes related to the JAK/STAT pathway that was shown to promote erythroid cell proliferation [3]. ETS2 and ERG are transcription factors that were shown to promote megakaryopoiesis while inhibiting erythroid maturation [5, 6]. Thus, genetic analyses suggest that loss of TP53, JAK/STAT overactivation, and aberrant expression of megakaryocytic transcription factors collaboratively promote the development of AEL.

Previous studies have reported several mouse models for AEL with *TP53* mutations or deletion [7]. Classically, loss of *Trp53* alleles or expression of mutant TP53 has been shown to promote the Friend virus- and Spi1-induced erythroleukemia [8, 9]. More recent models recapitulating the genetic alterations found in AEL have shown that *Trp53*-deficiency or *TP53* mutations cooperate with *JAK2-V617F, NTRK1-H498R, NFIA-ETO2*, and *ERG* to induce erythroleukemia in mice [4, 10–13]. Although these mouse models have provided insights into the pathogenesis of AEL, how these genetic alterations cooperate with TP53 inactivation in the process of erythroid transformation is still not fully understood. From a therapeutic point of view, it has been shown that JAK or Trk inhibitors are effective in inhibiting the development of AEL [3, 10]. However, such kinase inhibitors are often not sufficient to eliminate all the leukemic clones, and therefore other therapeutic targets in AEL need to be identified.

In this study, we investigated the effect of TP53 inhibition and ERG overexpression on erythroid leukemogenesis using human cord blood cells, human erythroid leukemia cell lines, and a mouse transplantation assay. TP53 inhibition promotes the growth of erythroid progenitors, while ERG inhibits terminal maturation of erythroid cells, and the combination induces the development of AEL. We also identified HDAC7 as a critical and specific regulator in erythroid leukemogenesis, which could be a promising therapeutic target for AEL.

## Methods

### Mice

C57BL/6 (Ly5.2) mice (Sankyo Labo Service Corporation, Tokyo, Japan) were used for bone marrow transplantation assays. *Trp53*^−/−^ mice, in which 5′ part of exon 2 including translation initiation site of *Trp53* gene was replaced with Neomycin resistance gene, were provided from the RIKEN BioResource Center (Ibaragi, Japan) [14]. Rosa26-LSL-Cas9 knockin mice were purchased from Jackson Laboratory (#024857) [15]. The *Trp53*^−/−^ mice were crossed with the Cas9 knockin mice to obtain *Trp53*^−/−^/Cas9 mice [16]. All animal experiments were approved by the Animal Care Committee of the Institute of Medical Science at the University of Tokyo (PA21–67) and were conducted following the Regulation on Animal Experimentation at University of Tokyo based on International Guiding Principles for Biomedical Research Involving Animals. We have complied with all relevant ethical regulations for animal testing.

### Cell culture

Human cord blood (CB) cells were obtained from the Japanese Red Cross Kanto-Koshinetsu Cord Blood Bank (Tokyo, Japan). Proper informed consent was obtained, and all experiments were performed according to an institutional review board‐approved protocol (approval number: 27‐34‐1225, 2023-3-0508), in accordance with the Declaration of Helsinki and The Belmont Report. Mono nuclear cells (MNCs) were isolated from CB by density gradient centrifugation using LymphoprepTM (density 1.077; Alere Technologies AS, Oslo, Norway). The CD34^+^ cell fraction was then isolated from the MNCs using the MidiMACS system (CD34^+^ Microbead Kit; Miltenyi Biotec; Bergisch Gladbach, Germany) according to the manufacturer’s protocols. CB CD34^+^ cells were incubated in StemSpanTM SFEMII (STEMCELL Technologies) supplemented with 100 ng/ml rhSCF (#255-SC, R&D Systems), 10 ng/ml rhIL-6 (#206-IL, R&D Systems), 1 ng/ml rhIL-3 (#203-IL, R&D Systems) and 1% penicillin–streptomycin (PS, #09367-34, Nacalai). CB cells were then transduced with vector or p53DD and were cultured in StemSpan SFEM II medium (#ST-09655, STEMCELL Technologies) with 2 U/ml erythropoietin (EPO, #3999412G7020, Kyowa Kirin). THP1 and Kasumi-1 cells were cultured in Roswell Park Memorial Institute (RMPI)-1640 medium (#189–02025, FUJIFILM Wako) with 10% fetal bovine serum (FBS; #FB-1365/500, Biosera) and 1% PS. F36P and TF-1 cells were cultured in RPMI-1640 medium supplemented with 10% FBS, 1% PS, and 2 ng/ml GM-CSF (#215-GMP, R&D Systems). cSAM cells were cultured in RPMI-1640 medium supplemented with 10% FBS containing 1 ng/ml mouse IL-3 (#203-IL, R&D Systems). CEP53 cells were cultured in RPMI-1640 medium supplemented with 10% FBS containing 1 ng/ml EPO (#959-ME, R&D Systems). Plat-E and 29T cells were cultured in Dulbecco’s Modified Eagle Medium (DMEM) medium (#044–29765, Wako) with 10% FBS and 1% PS.

### Plasmids

p53DD was obtained from Addgene (#25989) [17], and we cloned it into pMYs-IRES-NGFR vector [18]. MSCV-PIG, MSCV-PIG-ERG was obtained from Addgene (#66984) [19]. We added the AM-tag sequence (5′-TGCCAAGATCCTCAACGCAAAGGCAACGTGATACTCTCTCAGGCTTACGGGTGCCAAGATCCTCAACGCAAAGGCAACGTGATACTCTCTCAGGCTTACTAG-3′) into MSCV-PIG-ERG for the ChIP-Seq assay. pcDNA3-GATA1 (Addgene # 85693) [20], pSG5-hEKLF (Addgene #67835) [21] and pGL3-GATA-Luc (Addgene #85695) [20] were obtained from Addgene. pGL4.10[Luc2] (#E6651) and pGL4.74[hRluc/TK] (#E692A) were obtained from Promega. For pGL4.1-KLF1-Luc, we amplified the 6x repeat promoter sequence (5’-AGGGTGTGG-3’) of KLF1 [22] using PCR and inserted the PCR-amplified fragment into the restriction sites of pGL4.10[Luc2].

### Viral transduction

Retroviruses for mouse cells were generated by transient transfection of retroviral constructs into Plat-E packaging cells [23] using the calcium phosphate method. Retroviruses for human cells were generated by transient transfection of retroviral constructs along with M57 and RD114 into 293T cells using the calcium phosphate method. Retrovirus transduction to the cells was performed using Retronectin (Takara Bio Inc., Otsu, Shiga, Japan). Lentiviruses were produced by transient transfection of lentiviral plasmids along with pCMV-VSV-G (Addgene, #8454) [24] and psPAX2 (Addgene, #12260) into 293T cells using the calcium-phosphate method [25].

### Gene depletion using the CRISPR/Cas9 system

To generate short guide RNA (sgRNA) constructs, annealed oligos were inserted into pLentiguide-puro vector (#52963), pLKO5.sgRNA.EFS.tRFP657 vector (#57824) and pLKO5.sgRNA.EFS.GFP vector (#57822) [26], which were obtained from Addgene. Cas9 expressing vector was also obtained from addgene (lentiCas9-Blast #52962) [27]. Lentiviruses were produced by transient transfection of 293T cells as described above. Cells were infected with the virus for 24 h and were selected for stable expression of Cas9 using blasticidin (10 μg/ml). The sgRNA-transduced cells were selected using puromycin (1 μg/ml) or FACS-based sorting of tRFP657-positive cells.

For HDAC7 depletion in CB CD34^+^ cells, synthetic sgRNAs for sgHDAC7-A, sgHDAC7-B, and Cas9 protein were obtained from Integrated DNA Technologies (IDT). Cas9-sgRNA RNPs were produced by mixing 60 μM of synthetic sgRNA and 17 μg Cas9 for 30 min at room temperature. 3 × 10^5^ CB cells were electroporated in Buffer of P3 Primary Cell 4D-Nucleofector™ X Kit S (#V4XP-3032) using the 4D-Nucleofector system. Electroporation conditions used DZ-100.

For the HDAC7 and HDAC5 double knockout experiments in F36P cells, sgHDAC7 co-expressing tRFP657 and sgHDAC5 co-expressing GFP were transduced into the cells using lentivirus, and the frequency of GFP^+^ and/or tRFP657^+^ cells in the culture was monitored. For the HDAC7 and HDAC5 double knockout experiments in CEP53 cells, puroR sgHdac7 co-expressing puromycin-resistant gene (puroR) was first transduced into cells. After puromycin (1 μg/ml) selection, sgHdac5 co-expressing GFP was transduced into the cells and the frequency of GFP^+^ cells in the culture was monitored. The sequences of the sgRNAs are provided in Supplemental Table 1.

### Luciferase assay

1 × 10^5^ 293T cells were seeded in 12-well culture plates with cells in 500 μL medium. 18 h after seeding, the cells were transfected with pGL4.1-6X-KLF1 or pGL3-GATA-Luc (co-expressing Firefly Luciferase [FLuc]), pSG5-hEKLF (KLF1) or pcDNA3-GATA1, and pGL4.74 vector (co-expressing Renilla Luciferase [RLuc]) with MSCV-PIG-ERG or MSCV-PIG, using polyethylenimine (PEI). The cells were harvested 48 h after transfection and were assayed for the luciferase activity using the luciferase assay system (Promega) and a luminometer (BMG LABTECH, FLUOstar OPTIMA). Promoter activity was calculated as a ratio of Fluc to Rluc.

### Transplantation assay

Mouse bone marrow cells were collected from the Cas9 mice and *Trp53*^−/−^/Cas9 mice. Bone marrow progenitors (c-Kit^+^ cells) were selected using the CD117 MicroBead Kit (Miltenyi Biotec) and were pre-cultured in RPMI-1640 containing 10% FBS, 1% penicillin–streptomycin and 50 ng/ml murine SCF, 10 ng/ml TPO, 10 ng/ml IL-3 and 10 ng/ml IL-6 for 16 hr. These cells were then transduced with ERG and transplanted into sublethally (5.25 Gy) irradiated 12-week-old C57BL/6 mice. Each mouse received 2 × 10^5^ cells. For transplantation of CEP53 cells, each mouse received 5 × 10^6^ CEP53 cells without irradiation. For the first in vivo transplantation assay using sgRNAs targeting *Hdac7*, CEP53 cells were transduced with NT or a *Hdac7*-sgRNA (sg*Hdac7*-a) coexpressing tRFP657, and 5 × 10^5^ cells were transplanted into sublethally (525 cGy) irradiated 12 weeks-old C57BL/6 mice. We then collected bone marrow and spleen cells from the moribund mice, and compared the frequency of tRFP657^+^ (=sgRNA-transduced) cells in the GFP^+^ CEP53 cells. For the second transplantation assay, CEP53 cells were transduced with NT or a *Hdac7*-sgRNA (sg*Hdac7*-a) coexpressing puromycin-resistant gene (puroR), and 5×10^6^ cells were transplanted into sublethally (525 cGy) irradiated 12 weeks-old C57BL/6 mice to assess their survival.

### Flow cytometry

Cells were stained by fluoro-conjugated antibodies for 30 min at 4 °C. After staining, cells were washed with cold PBS two times and were resuspended in PBS containing 2% FBS. Cells were analyzed with Canto II (BD Biosciences, San Jose, CA, USA) and FlowJo software (FlowJo) or sorted with FACS Aria III (BD Biosciences, San Jose, CA, USA). The antibodies and their dilution ratios are provided in Supplemental Table 2.

### Western blotting

Cells were washed with PBS several times and lysed with pre-heated Laemmli sample buffer (Bio-rad, USA; #1610737). Total cell lysates were subjected to sodium dodecyl sulfate-polyacrylamide gel electrophoresis and transferred to a polyvinylidene fluoride membrane (Bio-Rad). Bands were visualized by LAS‐4000 Luminescent Image Analyzer (FUJIFILM). The antibodies and their dilution ratios are provided in Supplemental Table 3.

### RNA-seq

For RNA-seq with mouse AEL cells and erythroid progenitors, bone marrow cells were collected from normal C57BL/6 mice and the mice transplanted with *Trp53*^−/−^ERG-expressing cells. Cells were then stained with biotinylated Ter119 and CD71 antibodies and 2 × 10^6^ Ter119^+^CD71^+^ cells were sorted by AriaIII (BD Biosciences, San Jose, CA, USA). For RNA-seq with F36P cells, F36P cells were transduced with vector/ERG (coexpress GFP) or NT/HDAC7-sgRNAs. GFP^+^ cells were sorted by AriaIII. The sgRNA-transduced cells were selected with 1 μg/ml puromycin. Total RNA was extracted using RNeasy Mini Kit (Qiagen). After RNA fragmentation, cDNA was synthesized by random hexamer-primed reverse transcription, followed by a second-strand cDNA synthesis with dUTP. Following end repair, add A and adapter ligation, the DNA fragments were amplified by PCR. Libraries were sequenced using Illumina NovaSeq 6000 with paired-end mode (2 × 100 bp). Pair-end sequencing FASTQ files were aligned to the mouse reference genome (mm10). Raw gene counts were derived from the read alignments by Rsubread [28] (v2.12.3) and further transferred into count per million (CPM) by edgeR [29] (v3.40.2). After filtering out low-expression genes with CPM lower than 1, all CPM values were log2 transformed for generating unsupervised clustering dendrograms and heatmaps. Differential expression was analyzed with the linear model using limma [30] (v3.54.2). Genes with false discovery rate (FDR) < 0.05 adjusted by the Benjamini-Hochberg method were considered significant differentially expressing genes (DEGs) [31]. Pathway analyses were performed using GO Enrichment Analysis [32] and Gene Set Enrichment Analysis [33, 34]

### ChIP-seq

Chromatin immunoprecipitation was performed using Simple chip kit (Cell signaling technology, #9002) following the manufacturer’s instructions. 1 × 10^7^ F36P cells transduced with vector or ERG were fixed with 1% formaldehyde (Sigma) and then quenched with glycine. After washing and cell lysis, the chromatin was fragmented with 0.75 µl micrococcal nuclease (MNase) at 37 C for 15–20 min and nuclei were completely lysed by sonication. The 10 µg of chromatin in each reaction was incubated with 10 µl of anti-AM (#91111, Active motif), anti-H3K27ac (#8173, CST), or IgG antibody (#2729, CST) overnight at 4 C with rotation. Immunoprecipitation was performed with protein G magnetic beads. Following elution, reverse crosslinks, and purification, DNA was used for sequencing. ChIP-seq libraries were prepared and sequenced using Illumina Novaseq 6000 with paired-end mode (2 × 150 bp). Pair-end sequencing FASTQ files were aligned to the human reference genome (hg38) using Bowtie2 [35] on Galaxy platform (https://usegalaxy.org). Mapped reads were transformed by bamCoverage with the parameter “normalize using RPKM” from the deepTools [36]. Heatmap was generated by plotHeatmap which also from the deepTools. Peak calling was performed with MACS2 callpeak [37]. Gene list of peak calling was established by using R package CHIPseeker [38].

### Cell viability assay

The cytotoxicity of the class IIA HDAC inhibitor (TMP269) against various cell lines was assessed using the Cell Counting Kit-8 (Dojindo, Kumamoto, Japan) according to the provided instructions. Cells were plated at a density of 1 × 10^4^ cells/well in 0.1 ml of RPMI medium in 96-well plates and then treated with different concentrations of each compound. After 72 h of incubation with the compounds at 37 °C, 8 μl of Cell Counting Kit-8 solution was added to each well. Following 1 h incubation at 37 °C, absorbance at 450 nm was measured using a microplate reader (CLARIOstar Plus, BMG LABTECH, Ortenberg, GER). Relative cell viability was calculated as the ratio of the absorbance in each treatment group to that of the corresponding untreated control group. Data are presented as mean ± standard deviation (SD) of more than three independent experiments.

### Histone deacetylase enzyme activity measure assay

HDAC activity was evaluated using the HDAC-Glo^TM^ I/II Assay and Screening System (Promega #G6420). 1 × 10^4^ cells/well were seeded in a 96-well plate with 100 μL HDAC-Glo^TM^ I/II buffer. Then, 100 μL HDAC-Glo^TM^ I/II reagent and 1 μL developer reagent were added to each well. After incubation for 30 min at room temperature, luminescence was measured using the FLUOstar OPTIMA. Data are presented as mean ± standard deviation (SD) of more than three independent experiments.

### Statistical analyses

GraphPad Prism 9 was used for statistical analyses. Unpaired Student’s *t*-test (two-tailed) and Ordinary one-way ANOVA were used for pairwise comparisons of significance. The log rank (Mantel-Cox) was used for the survival curves comparison. Animal experiments were neither blinded nor randomized. The type of replication (biological or technical) is indicated in figure legends. Sample size was decided based on our previous experience in the field, not predetermined by a statistical method. All data are shown as mean ± SD.

## Results

### Distinct roles of TP53 and ERG in erythroid proliferation and differentiation

**Fig. 1:**
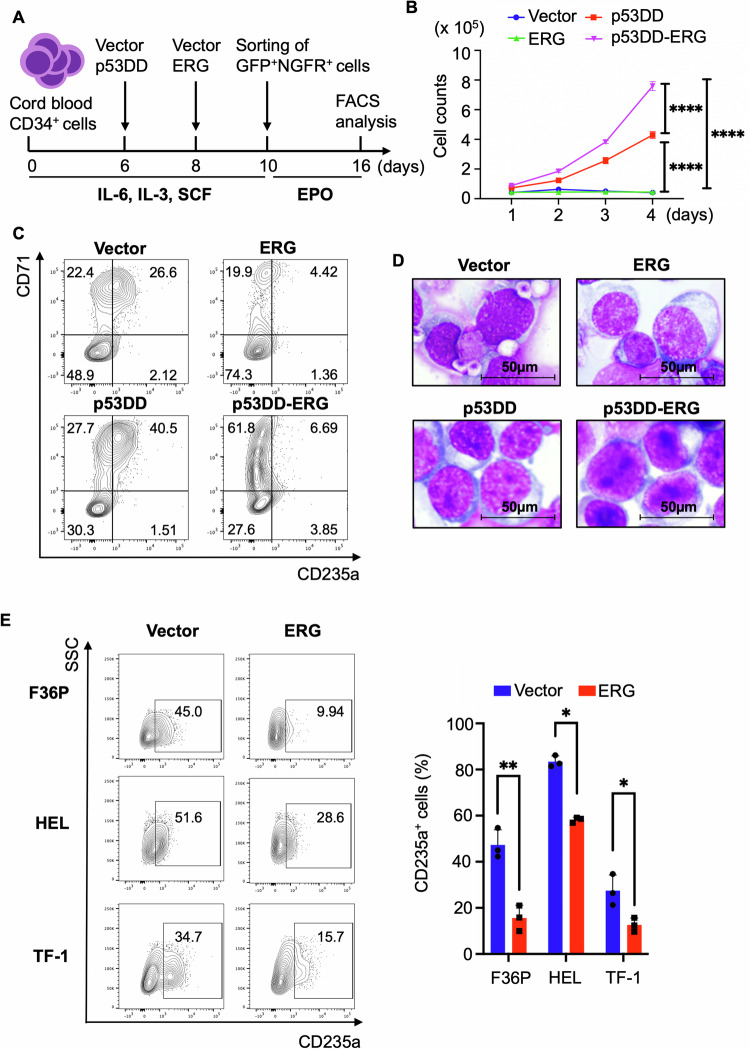
TP53 inactivation and ERG overexpression promote the growth of immature erythroid progenitors. **A** Experimental scheme used in (**B**–**D**). Human cord blood (CB) CD34^+^ cells were transduced with p53DD (coexpressing NGFR) and ERG (coexpressing GFP) and cultured in the presence of EPO. **B** Proliferation curves of CB cells transduced with vector, p53DD, ERG, or p53DD+ERG for 4 days. Results are expressed as mean ± s.e.m. of three independent experiments. ^****^P < 0.0001. CD71 and CD235a expression (**C**) and Wright-Giemsa-staining (**D**), original magnification x400) of CB cells transduced with vector, p53DD, ERG, or p53DD+ERG. **E** CD235a expression of F36P, HEL, and TF-1 cells transduced with vector or ERG.

We first assessed the effect of TP53 inhibition and ERG overexpression on proliferation and differentiation of human erythroid progenitors. We transduced ERG and/or dominant negative TP53 fragment (p53DD) into human cord blood (CB) CD34^+^ cells and cultured them in the presence of erythropoietin (EPO) to induce erythroid differentiation (Fig. 1A). p53DD promoted both proliferation and differentiation of CB cells, as evidenced by the increased cell numbers and CD71^+^CD235^+^ cells in culture (Fig. 1B, C). In contrast, ERG overexpression resulted in significant increase of CD71^-^CD235^-^ non-erythroid cells with little influence on CB cell proliferation. Notably, coexpression of ERG and p53DD promoted the efficient growth of immature CD71^+^CD235^−^ erythroid progenitors without enhancing erythroid differentiation (Fig. 1C). Morphological analysis revealed that the p53DD-transduced cells had visible dark nucleoli in the nucleus, indicating the effect of TP53 inactivation to increase erythroblasts (Fig. 1D). To further assess the role of ERG in erythroid differentiation, we transduced ERG into several human AML cell lines with erythroid properties: F36P, HEL, and TF-1. ERG expression was confirmed by western blotting (Supplemental Fig. 1A). The cells were then cultured with human EPO to induce erythroid differentiation for 6 days. Consistent with the earlier results, ERG overexpression inhibited the expression of an erythroid marker CD235a (glycophorin A) and the morphological changes induced by EPO in all these erythroid leukemia cell lines (Fig. 1E, Supplemental Fig. 1B). These data suggest a distinct role for TP53 and ERG in erythropoiesis: TP53 restricts proliferation and differentiation of hematopoietic stem and progenitor cells toward the erythroid lineage, whereas ERG inhibits terminal differentiation of erythroid progenitors. Importantly, TP53 inhibition together with ERG overexpression in human CB CD34^+^ cells induced rapid proliferation of immature erythroblasts.

### ERG inhibits erythroid differentiation by antagonizing KLF1 activity

**Fig. 2:**
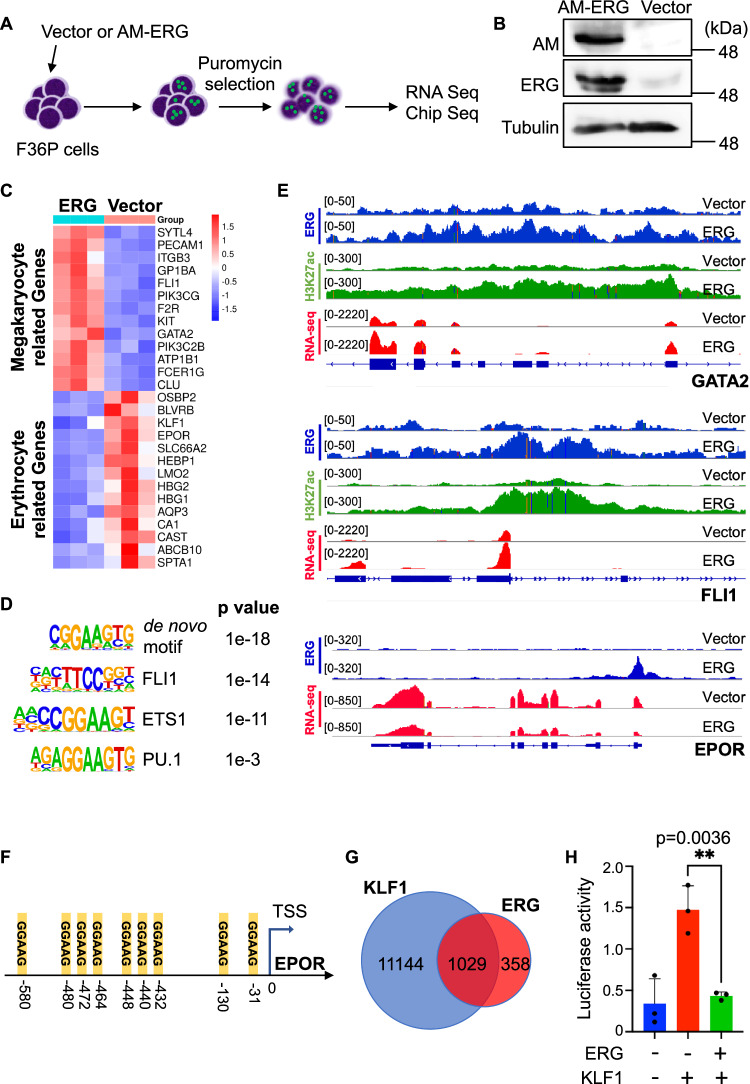
ERG represses erythroid gene expression and counteracts KLF1 activity. **A** Experimental scheme used in (**B**–**E**). **B** Western blotting of F36P cells transduced with vector or AM-tagged ERG. Cell lysates were stained with anti-AM, anti-ERG, or anti-α-Tubulin antibodies. **C** Expression levels of megakaryocyte- and erythrocyte-related genes in vector- or ERG-transduced F36P cells from RNA-seq data. **D** Top motifs of ERG binding sites from ChIP-seq data. **E** Integrative Genomic Viewer (IGV) screenshots of ChIP-seq (ERG: blue and H3K27ac: green) and RNA-seq (red) signals at *GATA2*, *FLI1*, and *EPOR* genes in vector- or ERG-transduced F36P cells. **F** Numerous ERG-binding sequences (GGAAG) are present in the promoter region of EPOR. **G** Venn diagram showing the overlap of ERG- and KLF1-bound genes in erythroid cells. **H** Reporter assay with the 6x KLF1 promoter in 293T cells. Results are expressed as mean ± s.e.m. of triplicates. ^**^P < 0.01.

To understand how ERG inhibits erythroid differentiation, we next performed RNA-Seq and ChIP-seq analyses using F36P cells expressing vector or ERG with a C-terminal AM (Active Motif) tag (Fig. 2A). High expression of AM-tagged ERG in F36P cells was confirmed by western blotting and RNA-seq (Fig. 2B, Supplemental Fig. 2B). RNA-seq revealed significant downregulation of several erythroid genes, including KLF1, EPOR, HBG and LMO2, in ERG-expressing F36P cells. In contrast, ERG overexpression induced upregulation of genes related to megakaryopoiesis, such as *ITGB3*, *GP1BA*, and *FLI1* (Fig. 2C, Supplemental Figs. 2A, B, Supplemental Dataset). Gene set enrichment analysis (GSEA) showed that ERG induced downregulation of GATA1-target genes and heme metabolism pathway genes, whereas it induced upregulation of genes related to megakaryopoiesis (Supplemental Fig. 2C). Thus, consistent with the earlier report [6], ERG overexpression promotes expression of megakaryocyte-specific genes while inhibiting erythroid gene expression.

ChIP-seq revealed that ERG bound to genomic regions containing the ETS core consensus sequence [5′-GGA(A/T)-3′], including those with FLI1, ETS1 and PU.1-binding sequences (Fig. 2D). More than 75% of ERG binding sites were located in promoter regions and many of them were enriched for histone 3 lysine 27 acetylation (H3K27ac), a marker of active transcription (Supplemental Fig. 2D, E, Supplemental Dataset). Genes associated with platelet activation, megakaryocyte and hemostasis pathways were upregulated by ERG with the increase of H3K27 acetylation at their promoters (Supplemental Fig. 2F). In particular, we identified GATA2 and FLI1 as direct ERG target genes in F36P cells (Fig. 2E). In addition to these ERG-activated genes, combined RNA-seq and ChIP-seq analyses led to the identification of EPOR as a potential repressive target of ERG (Fig. 2E). Indeed, the promoter region of EPOR contains an unusually large number of the ERG binding motif (GGAAG) (Fig. 2F).

Interestingly, more than 70% of the ERG-bound genes were also bound by KLF1 in erythrocytes (http://chip-atlas.org/target_genes public KLF1) (Fig. 2G). Given that KLF1 is a key transcription factor promoting erythropoiesis, these data suggest a possible competition between ERG and KLF1 to regulate erythroid gene expression. To test this possibility, we performed luciferase reporter assays using reporters containing the KLF1 (5′-AGGGTGTGG-3′) binding sequence. As expected, ERG repressed the transcriptional activity of the KLF1 promoter in HEK293T cells (Fig. 2H). Thus, similar to another ETS family member FLI1 [39], ERG inhibits erythroid maturation mainly by antagonizing KLF1 activity.

### *Trp53*-deficiency and ERG overexpression collaboratively promote the development of AEL

**Fig. 3:**
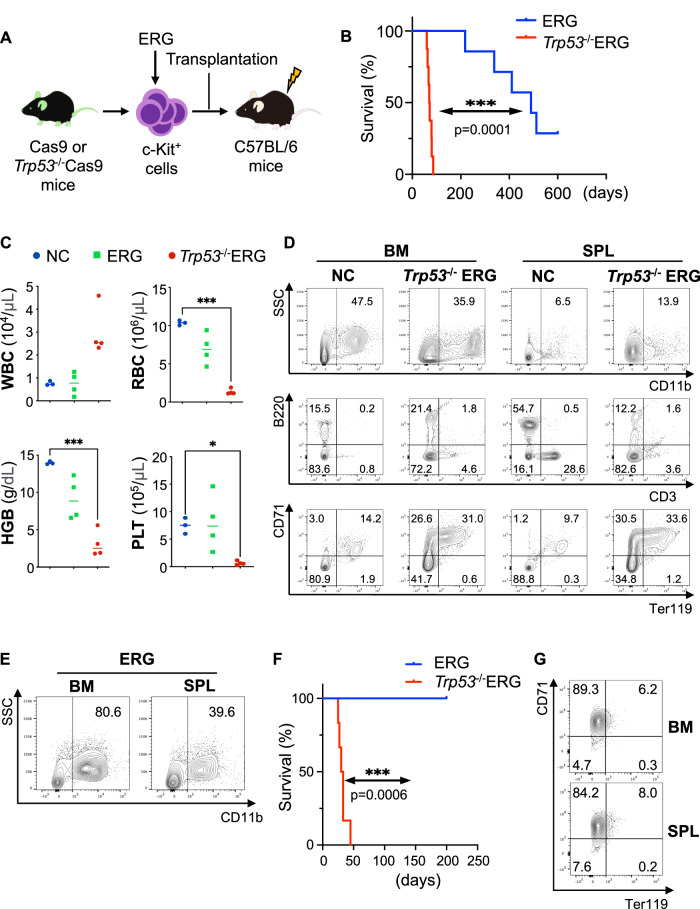
*Trp53* deficiency and ERG overexpression induce murine AEL. **A** Experimental scheme used in (**B**–**E**). **B** Kaplan–Meier survival curves of mice transplanted with ERG-expressing cells (*n* = 7) or *Trp53*^−/−^ERG-expressing cells (*n* = 8) are shown. **C** White blood cell (WBC), red blood cell (RBC), hemoglobin (Hb) and platelet (PLT) count in the negative control (NC: normal C57BL/6 mice, *n* = 3) mice and in the mice transplanted with ERG-expressing cells (ERG, *n* = 4) or *Trp53*^−/−^ERG-expressing cells (*Trp53*^−/−^ERG, *n* = 4) 8 weeks after transplantation. ^*^P < 0.05, ^***^P < 0.001. **D** Flow cytometric analysis of bone marrow (BM) and spleen (SPL) cells in the NC mice and mice transplanted with *Trp53*^−/−^ERG-expressing cells 8 weeks after transplantation. Quantified data are shown in Supplemental Fig. 3C. **E** Flow cytometric analysis of BM and SPL cells from mice transplanted with ERG-expressing cells 50 weeks after transplantation. **F** Kaplan–Meier survival curves of secondary recipient mice transplanted with ERG-expressing or *Trp53*^−/−^ERG-expressing spleen cells. **G** Flow cytometric analysis of BM and SPL cells in the secondary recipient mice transplanted with *Trp53*^−/−^ERG-expressing spleen cells 4 weeks after transplantation.

We next investigated the functional cooperation between ERG and *Trp53* (a murine homolog of TP53) deficiency in the development of AEL using a murine transplantation model. Bone marrow progenitor cells (c-kit^+^ cells) from 12 weeks *Trp53*^−/−^-Cas9 or Cas9 male mice were transduced with ERG, and 2 × 10^5^ cells were transplanted into sublethally (5.25 Gy) irradiated 8–12 weeks C57BL/6 male mice (Fig. 3A). All mice transplanted with Cas9^+^*Trp53*^−/−^ERG-expressing cells developed lethal leukemia approximately 60 days after transplantation (Fig. 3B). The mice bearing the Cas9^+^*Trp53*^−/−^ERG-expressing cells showed various hematopoietic abnormalities, including an increase in white blood cells, anemia and thrombocytopenia, while those receiving ERG-expressing cells showed only a trend toward mild anemia at two months post-transplantation (Fig. 3C). Morphological and immunophenotypic analyses of bone marrow and spleen cells revealed that the Cas9^+^*Trp53*^−/−^ERG-expressing cells induced marked increase of CD71^+^Ter119^+/−^ immature erythroblasts with concomitant decrease of B and T cells (Fig. 3D, Supplemental Fig. 3A, C). In contrast, ERG alone never caused rapid leukemia, but induced the development of neutrophilia after a long latency period (Fig. 3E, Supplemental Fig. 3B). We also transplanted the Cas9^+^*Trp53*^−/−^ERG-expressing spleen cells and the Cas9^+^ spleen cells expressing only ERG into secondary recipient mice. All mice transplanted with the Cas9^+^*Trp53*^−/−^ERG-expressing cells developed AEL within 45 days, whereas those receiving Cas9^+^ERG-expressing cells with intact *Trp53* did not (Fig. 3F, G). Thus, *Trp53*-deficiency and ERG overexpression are necessary and sufficient to fully transform c-kit^+^ adult bone marrow progenitors into AEL cells.

### Establishment of a mouse AEL cell line CEP53

**Fig. 4:**
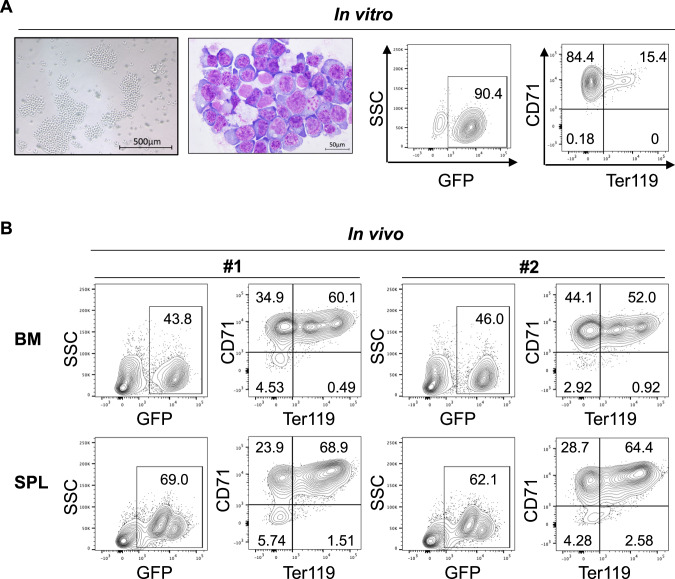
Establishment of a murine AEL cell line: CEP53. **A** CEP53 cells in suspension (far left), Wright-Giemsa staining of a cytospin slide of CEP53 cells (left) and flow cytometric analysis of CEP53 cells for GFP (right) and Ter119 and CD71 (far right). **B** CEP53 cells were transplanted into non-irradiated recipient mice. Bone marrow (BM) and spleen (SPL) cells were collected from two moribund mice. Expression of GFP, Ter119, CD71 in BM and SPL cells were shown. Note that almost all GFP^+^ CEP53 cells were CD71^+^Ter119^+/−^ erythroblasts.

To establish the Cas9-expressing murine AEL cell lines, we next cultured the AEL cells collected from a spleen of a moribund mouse bearing Cas9^+^*Trp53*^−/−^ERG-expressing cells in RMPI-1640 medium with various hematopoietic cytokines. SCF or IL-3 alone did not support the growth of spleen cells. SCF + IL-3 only promoted the growth of non-erythroid cells. In contrast, addition of EPO together with SCF and IL-3 promoted the marked expansion of GFP^+^ AEL cells in vitro (Supplemental Fig. 3D). The established AEL cells, we designated it CEP53 (Cas9 and ERG-expressing p53-deficient cells), grew well with 1 ng/ml EPO, expressed GFP as well as erythroid markers (CD71 and Ter119), and showed typical AEL morphology (dark nucleoli, increased nuclear/cytoplasmic ratio and cytoplasmic blebs) (Fig. 4A). To assess the repopulating capacity of CEP53, we transplanted 5 × 10^6^ CEP53 cells that had been cultured in vitro for 1 months into recipient mice without irradiation. All the mice developed AEL within 4 weeks, indicating that CEP53 cells retain the strong leukemogenicity even after the long-term in vitro culture (Fig. 4B, Supplemental Fig. 3E). Thus, we established a Cas9-expressing murine AEL cell line, CEP53, which can be cultured in vitro with only 1 ng/ml EPO, induces AEL in vivo even in unirradiated recipient mice, and allows efficient depletion of genes of interest using the CRISPR/Cas9 system.

### HDAC7 inhibits terminal maturation of human erythroid leukemia cells

**Fig. 5:**
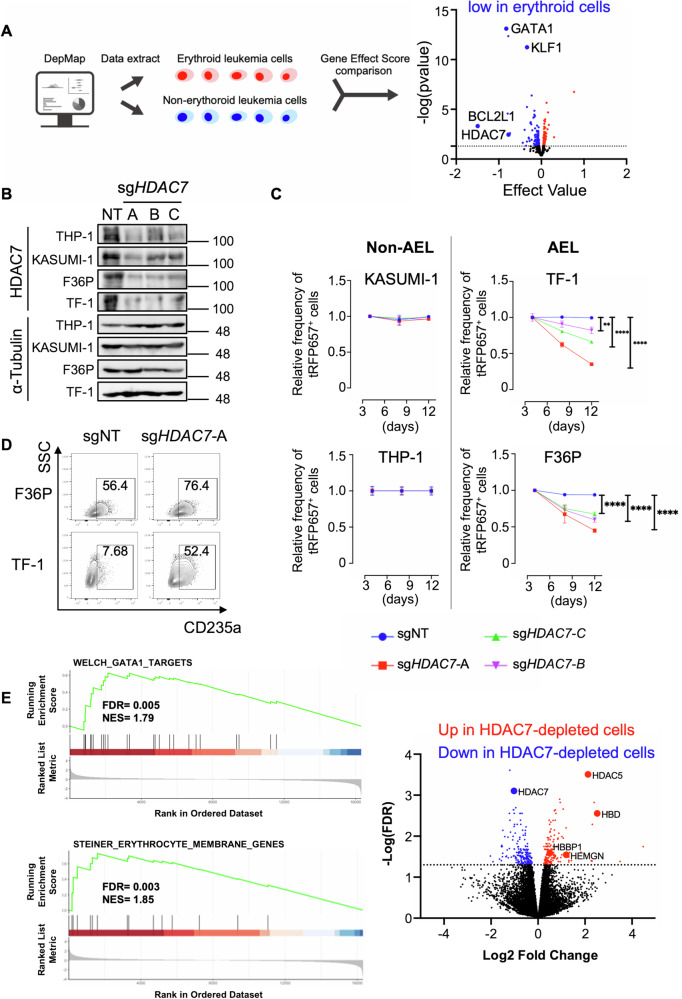
*HDAC7* depletion induces erythroid maturation in human AEL cells. **A** DepMap Gene Effect Score analysis between erythroid (*n* = 4) and non-erythroid human AML (*n* = 11). GATA1, KLF1, BCL2L1 and HDAC7 were selectively important for the growth of erythroid cells. **B** Erythroid (F36P and TF-1) and non-erythroid (THP-1 and Kasumi-1) cells were transduced with non-targeting (NT) or *HDAC7*-targeting (sg*HDAC7*-A/B/C) sgRNAs. The expression of HDAC7 and Tubulin in these cell lines was evaluated by Western blotting. **C** THP-1, KASUMI-1, F36P and TF-1 cells were transduced with the sg*HDAC7*-A/B/C or NT sgRNA co-expressing tRFP657 followed by in vitro cell culture. Results are normalized to the frequency of tRFP657^+^ cells at day 4, set to 1. Data are expressed as mean ± s.e.m. of three independent experiments. ^**^P < 0.01, ^****^P < 0.0001. **D** Flow cytometric analysis of CD235a expression in F36P and TF-1 cells transduced with sgNT or sg*HDAC7*-A transduction. **E** GSEA showing the enrichment of GATA-1 targets genes and erythrocyte membrane genes among the upregulated genes in *HDAC7*-depleted F36P cells (left). A volcano plot showing differentially expressed genes between control and *HDAC7*-depleted F36P cells. Down- (blue) and up- (red) regulated genes are defined by -log (FDR) greater than 1.3 (right).

Next, we applied the DepMap (https://depmap.org/portal/)-based two group comparison system (Nakahara, 2023, *GitHub*. Available at: https://github.com/jakushinn/depmap_analysis) to identify therapeutic vulnerabilities in AEL. We first divided 15 human AML cell lines into those with erythroid characteristics (HEL, F36P, TF-1 and OCIM2) and non-erythroid AML cells (KASUMI1, NB4, THP-1, U937, MV411, MOLM13, AML193, MOLM14, OCIAML2, OCIAML3, and MUTZ8). We compared the Gene Effect Score, which represents the essentiality of a gene in each cancer cell in DepMap, in the erythroid and non-erythroid leukemia cells. This analysis revealed several genes that are specifically important for the growth of erythroid leukemia cells, including BCL2L1, which was recently shown to be essential for the survival of erythroid/megakaryocytic AML [40] (Fig. 5A). We then integrated the DepMap analysis, RNA-seq data of mouse AEL cells and expression profiles of human AML patients and identified HDAC7 as a potential therapeutic target in AEL. HDAC7 is a class IIa histone deacetylase (HDAC) that is important for the growth of human erythroid leukemia cells, and is highly expressed in both human and mouse AEL (Fig. 5A, Supplemental Fig. 4A–C, Supplemental Dataset). To verify the role of HDAC7 in human AEL, we then assessed the effect of HDAC7 depletion in two erythroid (F36P and TF-1) and two non-erythroid (THP-1 and KASUMI-1) leukemia cell lines. We first transduced Cas9 (coexpressing Blasticidin S resistance gene: bsr) together with non-targeting (NT) or single-guide (sg)RNAs targeting *HDAC7* (coexpressing tRFP657) into these cells and monitored the frequency of tRFP657^+^ (HDAC7-depleted) cells in culture starting 96 h after the transduction. Efficient depletion of HDAC7 in these cells was confirmed by western blotting (Fig. 5B). Consistent with the data in DepMap, HDAC7 depletion suppressed the growth of erythroid leukemia cells but not that of non-erythroid leukemia cells (Fig. 5C). HDAC7 depletion promoted erythroid differentiation of TF-1 and F36P cells, as evidenced by the increased expression of CD235a, even in the absence of the EPO in culture (Fig. 5D). We also found that the ERG-mediated block of erythroid differentiation was partially reversed by HDAC7 depletion in F36P cells (Supplemental Fig. 4D). In addition, HDAC7 depletion promoted erythroid maturation of normal CB cells (Supplemental Fig. 4E), suggesting the key role of HDAC7 in the regulation of normal and malignant erythropoiesis.

Mechanistically, RNA-seq analysis revealed that loss of HDAC7 resulted in upregulation of several erythroid genes (*HBBP1*, *HBD* and *HEMGN*) and GATA1-target genes in F36P cells (Fig. 5E, Supplemental Dataset), confirming the enhanced erythroid maturation upon HDAC7 depletion. However, unlike ERG overexpression, HDAC7 depletion did not alter megakaryocytic gene expression, suggesting a more specific role of HDAC7 in erythroid differentiation. We also found that HDAC7 absence induced strong upregulation of *HDAC5*, another class IIa HDAC, in HDAC7-depleted F36P cells (Fig. 5E). However, depletion of HDAC5 showed no effect on proliferation and differentiation in F36P cells, even in those lacking *HDAC7*, indicating that HDAC5 does not have a redundant function to HDAC7 in AEL (Supplemental Fig. 5A). Thus, HDAC7 has the unique function to promote the growth of human erythroid leukemia cells by inhibiting their terminal maturation.

### Hdac7 promotes the development of mouse AEL

**Fig. 6:**
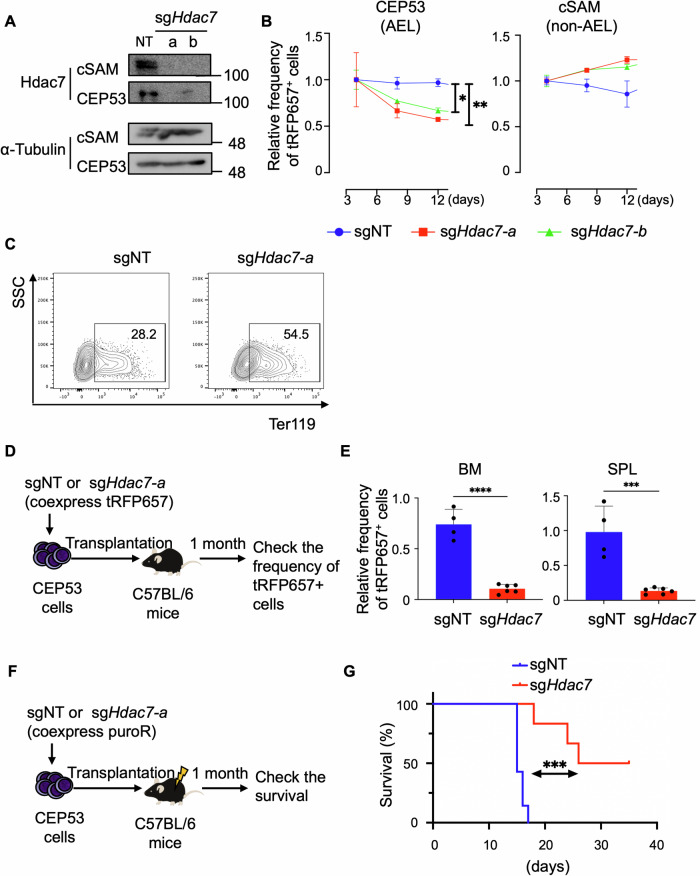
*Hdac7* deletion inhibits mouse AEL development. **A** CEP53 and cSAM cells were transduced with non-targeting (NT) or two independent *Hdac7*-targeting sgRNAs (sg*Hdac7*-a/b). The expression of Hdac7 and Tubulin in these cell lines was evaluated by Western blotting. **B** CEP53 and cSAM cells were transduced with NT or sg*Hdac7*-a/b co-expressing tRFP657 followed by in vitro cell culture. Results are normalized to the frequency of tRFP657^+^ cells at day 4, set to 1. Data are expressed as mean ± s.e.m. of three independent experiments. ^*^P < 0.05, ^**^P < 0.01. **C** Flow cytometric analysis of Ter119 in control or *Hdac7*-depleted CEP53 cells. **D** Experimental scheme used in (**E**). CEP53 cells were transduced with sgNT or sg*Hdac7*-*a* co-expressing tRFP657, following transplantation into non-irradiated recipient mice. Representative FACS plots are shown in Supplemental Fig. 5A. **E** Relative ratios of the tRFP657^+^ (sgRNA-transduced) fraction in GFP^+^ CEP53 cells after transplantation compared to that before transplantation are shown as mean ± s.e.m. ^***^P < 0.001, ^****^P < 0.0001. sgNT *n* = 4, sgHdac7 *n* = 6 for each group. **F** Experimental scheme used in (**G**). CEP53 cells were transduced with sgNT or sg*Hdac7*-*a* co-expressing puromycin-resistant (puroR) gene, following transplantation into Sub-irradiated recipient mice to assess their survival. **G** Kaplan–Meier survival curves of mice transplanted with sgNT (*n* = 7)　or sg*Hdac7*　(*n* = 6) transduced CEP53 cells. ^***^P < 0.001, log-rank test.

We next assessed the role of HDAC7 in the mouse AEL cell line, CEP53. We transduced NT or sgRNAs targeting mouse *Hdac7* (coexpressing tRFP657) into CEP53 and monitored the frequency of tRFP657^+^ (*Hdac7*-depleted) cells in culture. Similar to the earlier results obtained with the human erythroid leukemia cells, *Hdac7* depletion inhibited the growth of CEP53 cells in vitro by inducing their erythroid differentiation. In contrast, loss of HDAC7 did not inhibit the growth of cSAM cells [41], the mouse monocytic AML cells transformed by SETBP1 and ASXL1 mutations (Fig. 6A–C). In addition, HDAC5 depletion did not change the proliferation and differentiation of CEP53 cells **(**Supplemental Fig. 5B). These data confirm the selective role of HDAC7 in erythroid leukemia. To determine the role of HDAC7 in the development of AEL in vivo, we then performed transplantation assay using CEP53 cells transduced with NT or the *Hdac7*-targeting sgRNA (sg*Hdac7*-a) coexpressing tRFP657. We collected GFP^+^ leukemia cells from bone marrow and spleen 25 days after transplantation, at which time all mice developed AEL. We observed substantial decrease of tRFP657^+^ (*Hdac7*-depleted) cells in both bone marrow and spleen, indicating the critical role of HDAC7 in promoting the in vivo development of AEL (Fig. 6D, E, Supplemental Fig. 6). We next performed a similar experiment using sgNT and sg*Hdac7*-a, which coexpress a puromycin-resistant gene. After puromycin selection, the sgNT or sg*Hdac7*-transduced CEP53 cells were transplanted into recipient mice to assess their survival. As expected, Hdac7 depletion significantly prolonged the survival of leukemic mice bearing CEP53 cells (Fig. 6F, G). Taken together, we concluded that HDAC7 is a critical regulator in human and mouse AEL.

### Enzymatic function of HDAC7 is dispensable for the growth of AEL

**Fig. 7:**
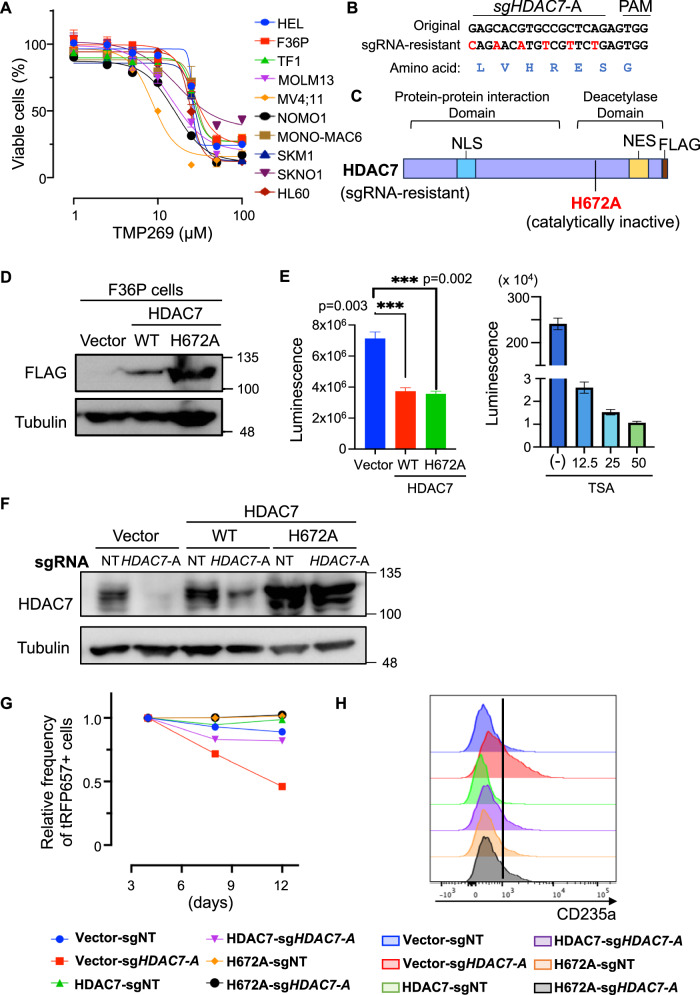
HDAC7 promotes AEL cell growth through non-enzymatic function. **A** Erythroid (HEL, F36P, TF-1) and non-erythroid (MOLM13, MV4;11, NOMO1, MONO-MAC6, SKM1, SKNO1, HL60) human AML cell lines were treated with TMP269 (1–100 μM) for 72 h in three technical replicates. **B** Construction of sgRNA-resistant HDAC7 cDNA. **C** Schematic representation of HDAC7. The H672A mutation was shown to abolish its enzymatic activity. **D** F36P cells were transduced with vector, FLAG-tagged wild-type (WT) HDAC7 or HDAC7-H672A, and their expression was confirmed by Western blotting. **E** The dose-dependent reduction of HDAC activity in F36P cells by TSA treatment was confirmed using the same assay (left). HDAC activity was evaluated in vector, HDAC7-WT or HDAC7-H672A-transduced F36P cells using the HDAC-Glo^TM^ I/II assay (right). **F** The vector, HDAC7-WT or HDAC7-H672A transduced F36P cells were then transduced with non-targeting (NT) or *HDAC7*-targeting (sg*HDAC7*-A) sgRNA. HDAC7 and Tubulin expression was evaluated by Western blotting. **G** The vector, HDAC7-WT or HDAC7-H672A transduced F36P cells were transduced with NT or sg*Hdac7*-A co-expressing tRFP657 followed by in vitro cell culture. Results are normalized to the frequency of tRFP657^+^ cells at day 4, set to 1. **H** Flow cytometric histograms of CD235a expression in F36P cells transduced with vector-, HDAC7-WT or HDAC7-H672A together with NT or sg*Hdac7*-A.

Next, we assessed the effect of a selective Class IIa HDAC inhibitor TMP269 on various human AML cell lines including AEL. However, contrary to the expectation, AEL cell lines were not more sensitive to TMP269 than non-AEL cell lines (Fig. 7A). We therefore hypothesized that the HDAC7 may promote the growth of AEL cells through non-enzymatic functions. To test this hypothesis, we first designed sgRNA-resistant cDNA by introducing synonymous mutations in the sgRNA-target sequences to prevent the recognition of *HDAC7*-targeting sgRNA (sg*HDAC7*-A) (Fig. 7B). We then introduced a histidine (H) to alanin (A) mutation at H672 in human HDAC7 to generate a catalytically inactive HDAC7 mutant [42] (Fig. 7C). We transduced vector, wild-type (WT) HDAC7 or HDAC7-H672A into F36P cells and assessed the HDAC activity in them (Fig. 7D). The HDAC enzyme inhibitor trichostatin A (TSA) showed the expected dose-dependent reduction in HDAC enzyme activity. However, both wild-type and mutant HDAC7 did not enhance but rather inhibited the HDAC activity in F36P cells (Fig. 7E), suggesting the possibility that HDAC7 may not act as a “histone deacetylase” in erythroid leukemia cells.

We then transduced NT or HDAC7-targeting sgRNAs into F36P cells expressing vector or sgRNA-resistant HDAC7 constructs. Successful transduction of HDAC7 constructs and sgRNA-mediated depletion of endogenous HDAC7 were confirmed by western blotting (Fig. 7F). As expected, HDAC7 depletion induced erythroid differentiation and inhibited the growth of F36P cells, which was canceled in the wild-type HDAC7-transduced F36P cells. Importantly, the catalytically inactive HDAC7 mutant also reversed the effect of HDAC7 depletion on the growth and maturation of F36P cells as efficiently as wild-type HDAC7 (Fig. 7G, H, Supplemental Fig. 5B). Collectively, these results suggest that the HDAC7 enzymatic activity is dispensable for the cell growth and differentiation in AEL.

## Discussion

ERG is a versatile oncogene that has been shown to induce the development of various types of leukemia with additional genetic alterations. Our data together with previous reports clearly showed that TP53 inactivation cooperates with ERG to induce the development of erythroleukemia [5, 43–45]. However, most previous studies have used fetal liver-derived erythroblasts, which are more likely to recapitulate pediatric AEL. In this study, we demonstrate that the combination of ERG overexpression and *Trp53*-deficiency can transform c-kit^+^ adult bone marrow progenitors into AEL cells, providing a simple experimental model for adult AEL.

Mechanistically, ERG interferes with erythroid differentiation by antagonizing KLF1 activity, while promoting upregulation of megakaryocytic genes. This function of ERG is similar to that of another ETS transcription factor, FLI1, which has been shown to play a critical role in the megakaryocytic/erythroid bifurcation by counteracting KLF1 [46]. Another interesting finding is the unexpected ERG-mediated suppression of EPOR, given that EPOR amplification frequently coexists in patients with AEL harboring *TP53* mutations and ERG amplification [3, 47]. This seemingly contradictory finding suggests that the elevated expression of EPOR and the consequent activation of the JAK pathway need to be attenuated by ERG for leukemic transformation. Like ERG, a recent study showed that NFIA-ETO2 fusion blocks erythroid maturation and induces AEL in cooperation with mutant TP53 [48]. Thus, suppression of erythroid gene expression programs by transcription factors appears to be necessary to generate fully transformed AEL in cooperation with TP53 inactivation. To inhibit TP53 function, we used p53DD overexpression in human CB CD34^+^ cells and *Trp53*^−/−^ mice to establish the mouse AEL model. Since both approaches similarly enhanced erythroid cell proliferation, it is likely that TP53 loss-of-function, rather than gain-of-function, promotes the development of AEL.

Given its multiple roles, including its essential role in hematopoietic stem cell maintenance, ERG may not be the ideal therapeutic target in AEL. Instead, we identified HDAC7 as a selective regulator in erythroleukemia. HDAC7 is a member of the class IIa family of HDACs (HDAC4/5/6/7), which intrinsically possess low enzymatic activity but harbor a unique adapter domain in the N-terminus that mediates binding to several transcription factors [49]. Importantly, we have shown that HDAC7 maintains the growth of AEL cells through non-enzymatic functions. Therefore, it is necessary to develop HDAC7 degraders rather than the deacetylase inhibitor to treat AEL. The mechanisms by which HDAC7 promotes AEL development also need to be elucidated in future studies. In addition to HDAC7, a previous study showed that another class IIa HDAC, HDAC5, forms a complex with GATA1 and KLF1 to regulate normal erythroid maturation [50]. However, HDAC5 is dispensable for the growth of human and mouse AEL cells, suggesting that HDAC7 has a unique non-enzymatic function to promote the leukemogenicity of AEL. In addition to malignant erythropoiesis, our data suggest that HDAC7 is also involved in normal erythroid maturation. The precise role of HDAC7 in normal hematopoiesis warrants further investigation.

In summary, we showed the distinct and cooperative functions of TP53 loss and ERG overexpression during erythroid transformation. We also established a novel mouse model of adult AEL that will be useful to evaluate the role of specific genes or to test the effect of drug candidates in a physiological microenvironment with a functional immune system. In addition, our study highlights HDAC7 as a promising therapeutic target in AEL that is resistant to current standard therapies.

## Supplementary information

Supplemental Figures

Supplemental Table

Supplemental Dataset

## Data Availability

The datasets generated during and/or analyzed during the current study are available from the corresponding author on reasonable request.
