# Supplementary material for: HDAC7 is a potential therapeutic target in acute erythroid leukemia
Source: Leukemia. 2024 Sep 15;38(12):2614–27. doi: 10.1038/s41375-024-02394-5 (PMC11588653; doi:10.1038/s41375-024-02394-5)

**Supplemental Figures**

**Supplemental Figure legends**

**Supplemental Figure 1. ERG overexpression in human AEL cell lines.**

**A.** F36P, HEL and TF-1 cells were transduced with vector or ERG. Expression of ERG and GAPDH was confirmed by Western blotting. **B.** F36P, HEL and TF-1 cells transduced with vector or ERG were cultured in the presence of EPO for 6 days. Wright-Giemsa staining of the cytospins is shown. Original magnification x400.

**Supplemental Figure 2. Gene expression and epigenetic changes induced by ERG overexpression in F36P cells.**

**A.** Heatmap of the top 200 differentially expressed genes between F36P cells transduced with vector or ERG. **B.** Volcano plot of differentially expressed genes between vector and ERG-transduced F36P cells. Down- (blue) or up- (Red) regulated genes in ERG-expressing F36P cells are defined by -log(FDR) greater than 1.3. **C.** GSEA showing the enrichment of megakaryocyte related genes among the upregulated genes, and the enrichment of GATA1-target genes and genes associated with heme metabolism among the downregulated genes in ERG-expressing F36P cells. **D.** Heatmap of ERG binding and H3K27ac peaks that are gained or lost in ERG-expressing F36P cells. **E**. Peak distribution of ERG and H3K27ac enriched sites. **F.** Venn diagram showing the overlap of H3K27ac-enriched genes and upregulated genes in ERG-expressing F36P cells (left). Among the 82 overlapping genes, those related to platelet activation, megakaryocytes, and the hemostasis pathway are listed in a table (right).

**Supplemental Figure 3. *Trp53* deficiency combined with ERG overexpression induces the development of murine AEL.**

**A.** Wright-Giemsa staining of cytospins from bone marrow (BM) and spleen (SPL) cells collected from negative control (NC = normal C57BL/6) mice and the mice transplanted with *Trp53*^-/-^ERG-expressing cells 8 weeks after transplantation. Magnification, x100. **B.** Wright-Giemsa staining of cytospins from BM and SPL cells obtained from the mice transplanted with ERG-expressing cells 50 weeks after transplantation. Magnification, x100. **C.** Frequency of B220^+^ B cells (left), CD3^+^ T cells (middle) and CD71^+^Ter119^+/-^ erythroblasts (right) in bone marrow (BM) and spleen (SPL) of NC mice and those transplanted with the *Trp53*^-/-^ERG-expressing cells. n=3 for each. Results are expressed as mean ± s.e.m. ^*^*P*<0.05, ^**^*P*<0.01, ^***^*P*<0.001. Representative FACS plots are shown in Figure 3D. **D.** Flow cytometric analysis of the mouse AEL cells cultured with different cytokines. The GFP^+^CD71^+^Ter119^+/-^ AEL cells were expanded only in the EPO-containing culture. **E.** Kaplan–Meier survival curves of recipient mice transplanted with CEP53 cells without irradiation. (n=6)

**Supplemental Figure 4. HDAC7 is a critical regulator in AEL.**

**A.** (Left) CD71^+^Ter119^-/+^erythroid precursor cells were obtained from negative control (NC: normal C57BL/6) mice and the mice transplanted with *Trp53*^-/-^ERG-expressing cells. Representative FACS plots are shown. (Right) Volcano plot of showing differentially expressed genes between normal and malignant erythroid precursor cells. Up- (red) or down- (blue) regulated genes in AEL cells are defined by -log(FDR) greater than 1.3. **B**. Venn diagram showing the overlap of the top 100 upregulated genes in mouse AEL cells compared to normal erythroid precursors and the top 100 essential genes in human erythroid leukemia cells revealed by the DepMap data. We identified 3 genes (HDAC7, FNBP1L, and SPINT2) that are highly expressed in mouse AEL and important for the growth of human erythroid leukemia cells. **C**. Among them, HDAC7 is also highly expressed in AEL cells from an M6 patient. Data were collected from cBioportal (https://www.cbioportal.org) data were used in these analyses. **D.** F36P cells were transduced with vector or ERG, and then transduced with non-targeting (NT) or HDAC7-targeting (sgHDAC7-A) sgRNA. CD235a expression in these cells was assessed after 6 days of culture with EPO. Representative FACS plots (left) and their quantification (right). Results are expressed as mean ± s.e.m. of three independent experiments. ^****^*P*<0.0001. **E**. (left) Human cord blood (CB) CD34^+^ cells were transduced with sgNT, sg*HDAC7A* or *B*. Expression of HDAC7 and α-Tubulin in these CB cells was evaluated by Western blotting. (right) Expression of erythroid markers (CD71 and CD235a) in these cells was assessed after 6 days of culture with EPO. Note that HDAC7 depletion increased the frequency of CD235^+^CD71^-^ mature erythroid cells.

**Supplemental Figure 5. No functional redundancy between HDAC5 and HDAC7 in AEL.**

**A.** (left top) F36P cells were transduced with sgNT or sg*HDAC5*. Expression of HDAC5 and α-Tubulin was evaluated by Western blotting. (left bottom) F36P cells were transduced with sgNT or sg*HDAC7* (co-expressing tRFP657) together with sgNT or sg*HDAC5* (co-expressing GFP) followed by *in vitro* cell culture. Results are normalized to the frequency of tRFP657^+^ and/or GFP^+^ cells at day 4, set to 1. Data are expressed as mean ± s.e.m. of three independent experiments. ^****^*P*<0.0001. (right) Flow cytometric analysis of CD235a expression in sgNT, sg*HDAC5*, sg*HDAC7,* or sgHDAC5/sgHDAC7-transduced F36P cells. **B.** (left top) CEP53 cells were transduced with sgNT and sg*Hdac5*. Expression of HDAC5 and α-Tubulin was evaluated by Western blotting. (left bottom) CEP53 cells were transduced with sgNT or sg*Hdac7* co-expressing a puromycin resistant gene together with sgNT or sg*Hdac5* co-expressing GFP, followed by *in vitro* cell culture. Results are normalized to the frequency of GFP^+^ cells at day 4, set to 1. Data are expressed as mean ± s.e.m. of three independent experiments. (right) Flow cytometric analysis of Ter119 expression in sgNT, sg*HDAC5*, sg*HDAC7,* or sgHDAC5/sgHDAC7-transduced CEP53 cells.

**Supplemental Figure 6. HDAC7 promotes the growth of AEL cells *in vivo* and *in vitro* through non-enzymatic activity.**

**A.** CEP53 cells were transduced with non-targeting (NT) or *Hdac7*-targeting (sg*Hdac7*-a) sgRNA co-expressing tRFP657. These cells were transplanted into recipient mice and the frequency of tRFP657^+^ cells among the GFP^+^ CEP53 cells in bone marrow (BM) and spleen (SPL) was assessed 4 weeks after transplantation. Representative FACS plots before and after transplantation are shown. Quantified data are shown in Figure 6E. **B.** Independent repeat experiment of Figure 7G. F36P cells were transduced with vector, HDAC7-WT or HDAC7-H672A together with NT or sg*HDAC7*-A (co-expressing tRFP657) followed by *in vitro* cell culture. Results are normalized to the frequency of tRFP657^+^ cells at day 4, set to 1.


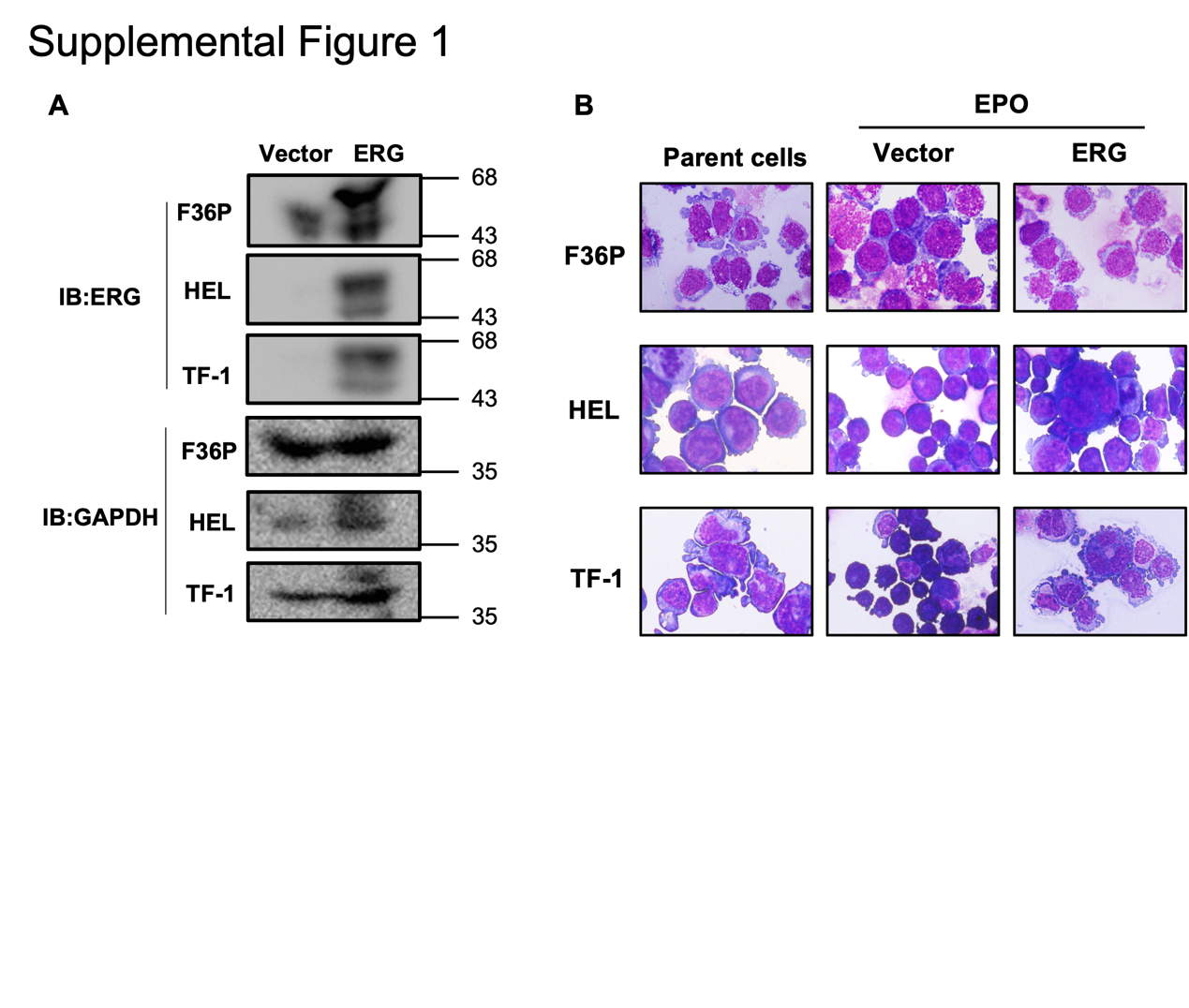

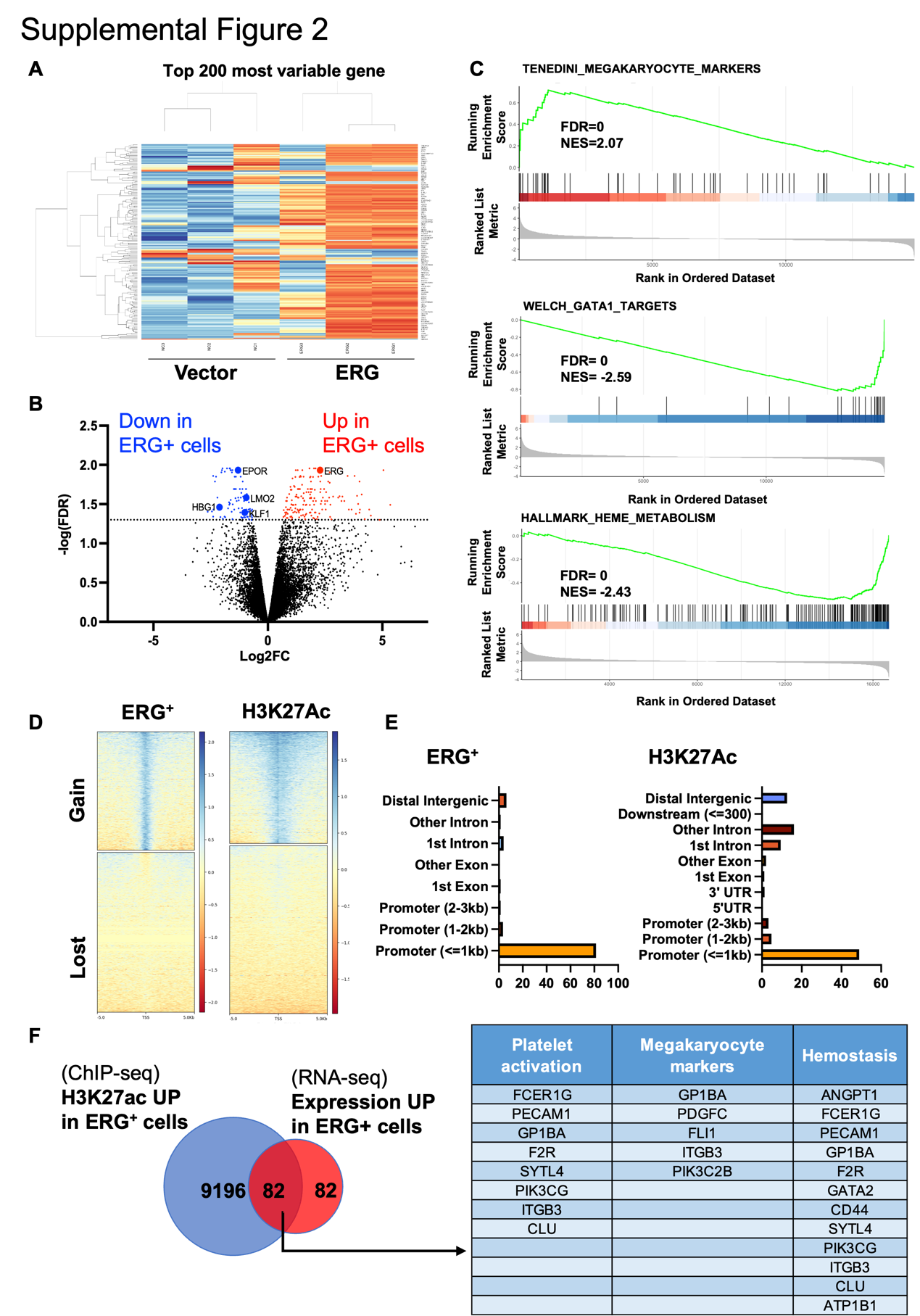

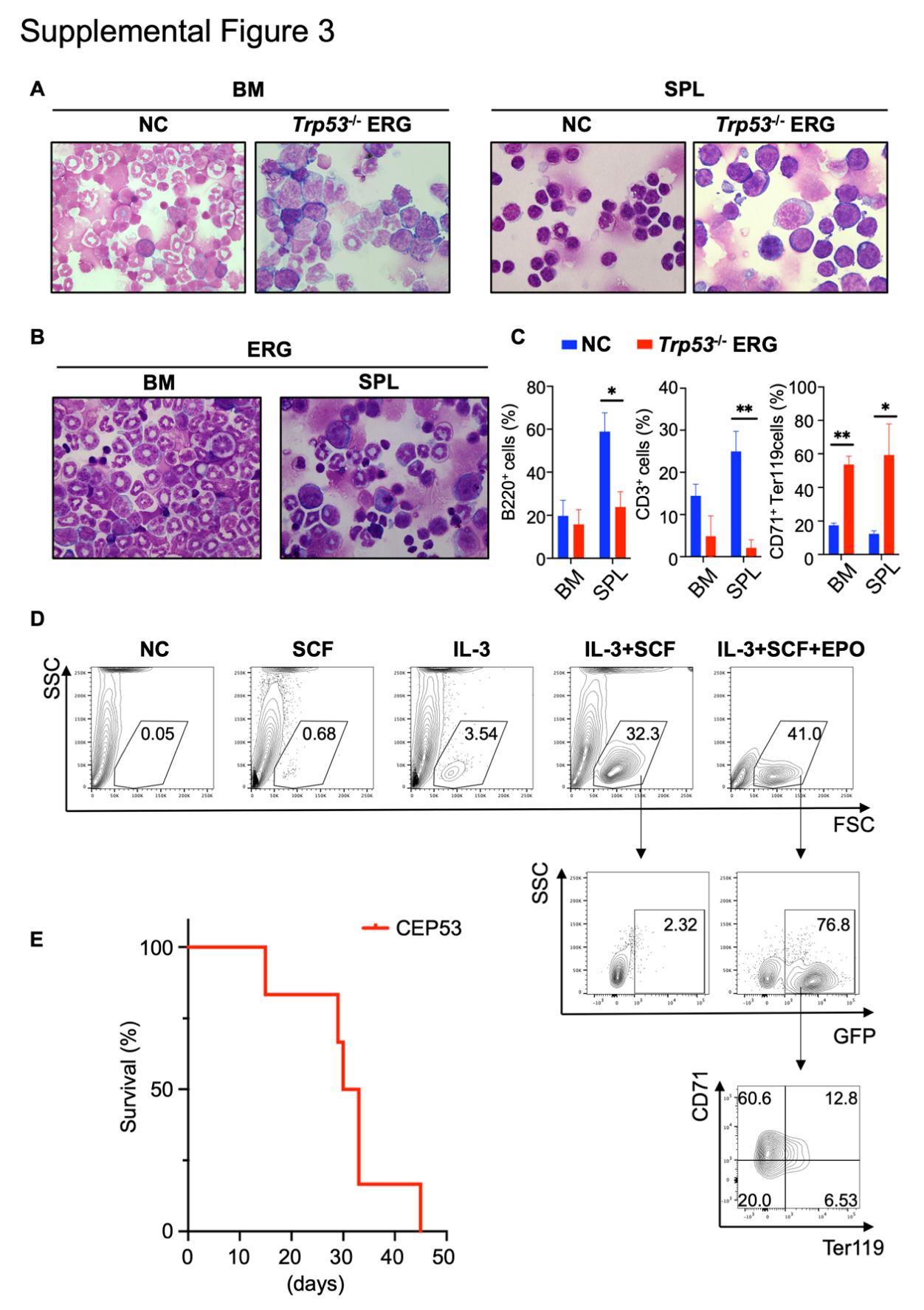

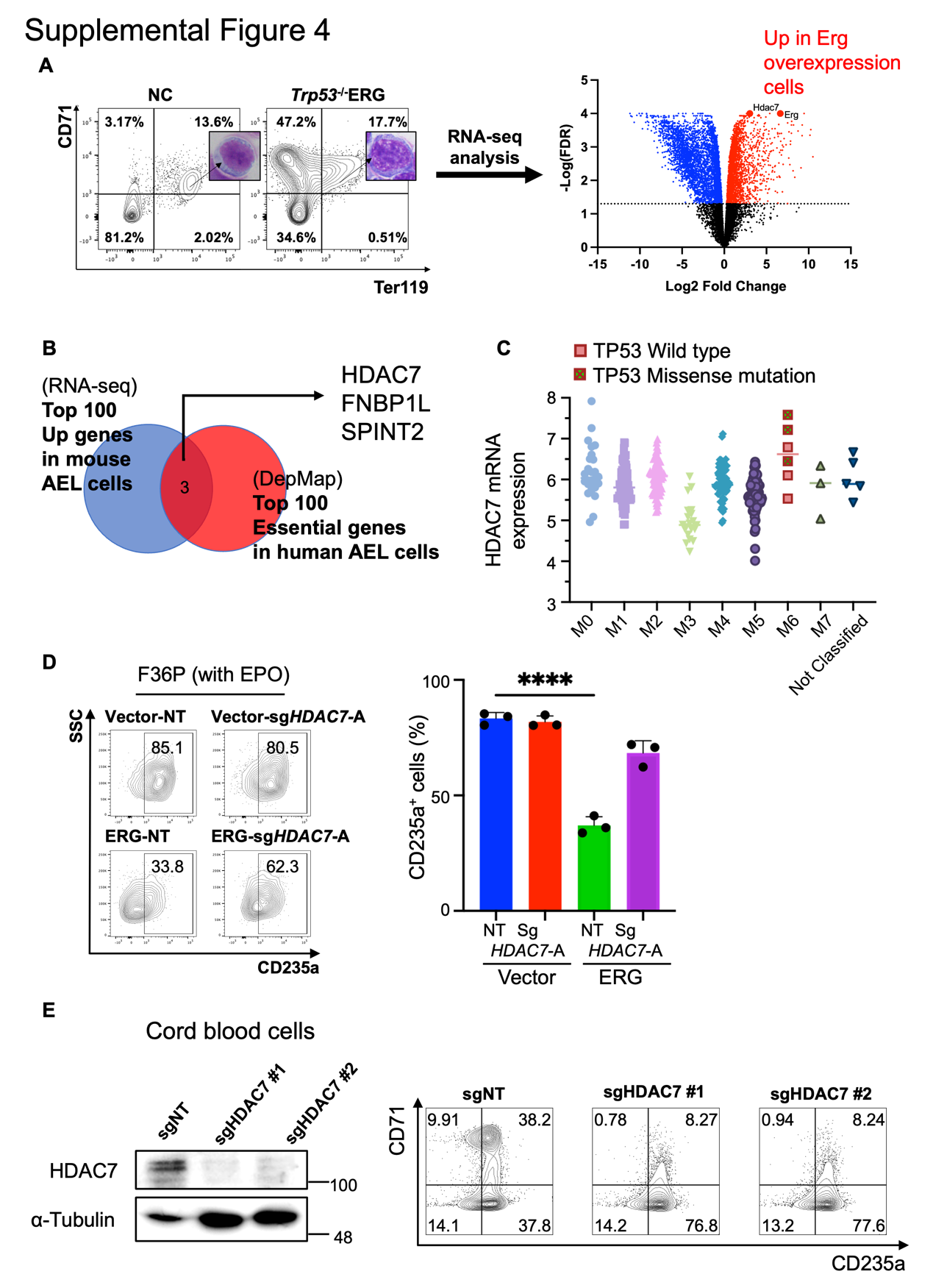

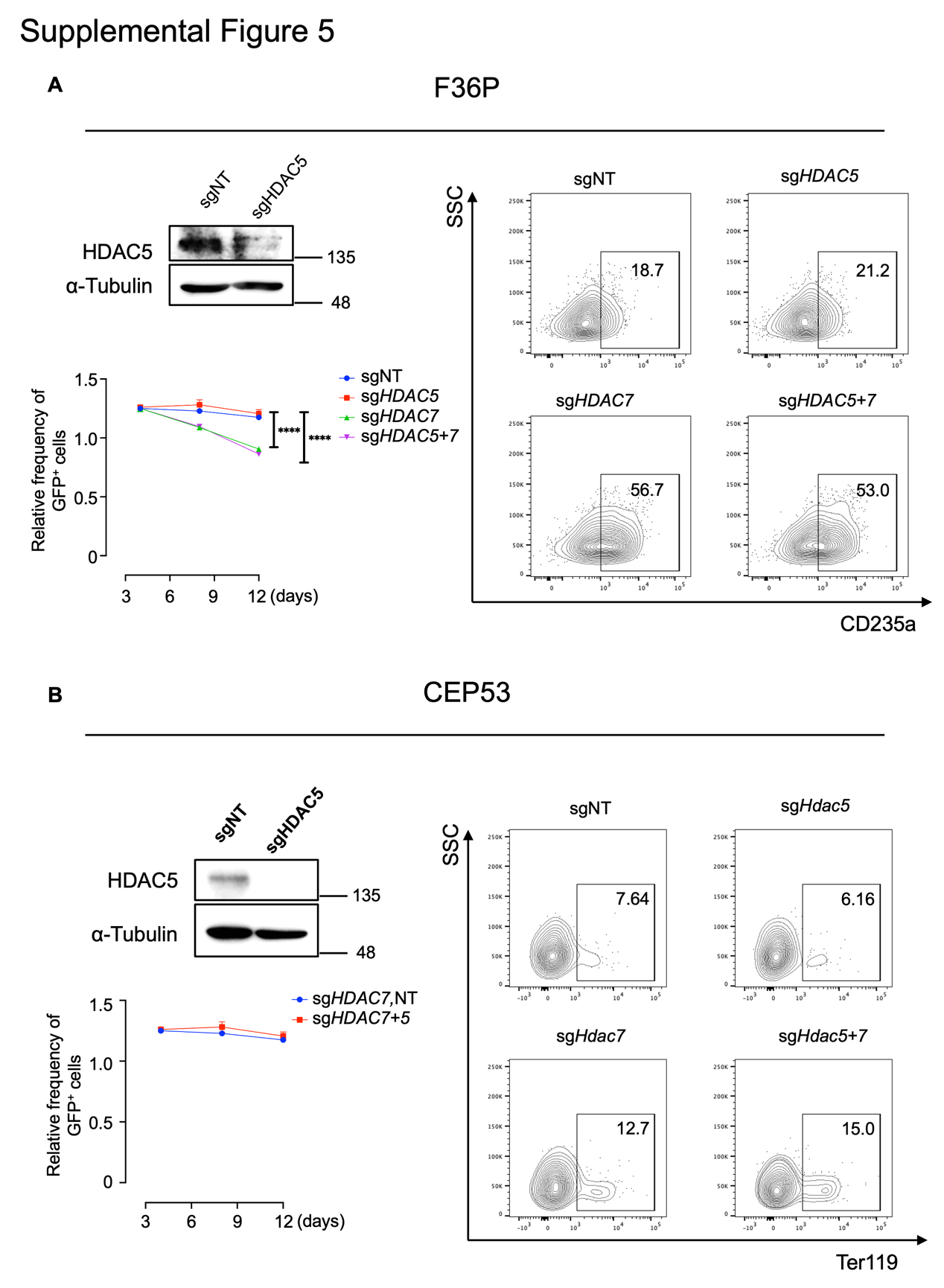


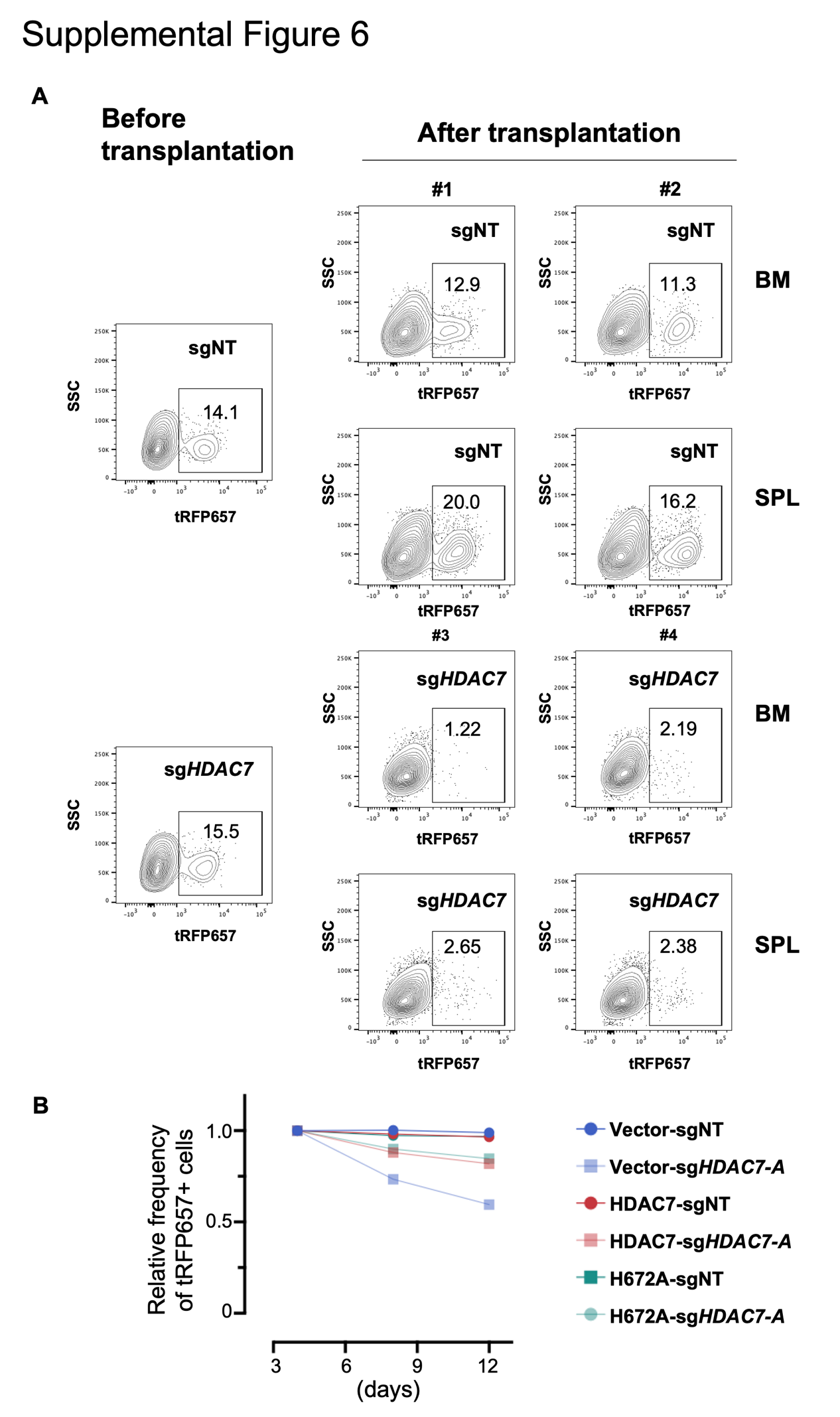

Supplement: Supplementary file 1 — Supplemental Figures [file 41375_2024_2394_MOESM1_ESM.docx]
