# Supplementary material for: HDAC7 is a potential therapeutic target in acute erythroid leukemia
Source: Leukemia. 2024 Sep 15;38(12):2614–27. doi: 10.1038/s41375-024-02394-5 (PMC11588653; doi:10.1038/s41375-024-02394-5)
Supplement: Supplementary file 2 — Supplemental Table [file 41375_2024_2394_MOESM2_ESM.docx]

**Supplemental Table 1**

**sgRNA sequences**

**Supplemental Table 2**

**Antibody List for flow cytometry**

**Supplementary Table 3**

**Antibody List for western blotting**
